# Children co-victims of intimate partner homicide: the first 72 decisive hours

**DOI:** 10.3389/fpubh.2025.1494289

**Published:** 2025-06-05

**Authors:** Nathalie Prieto, Karine Zurcher, Léa Guyot, Perrine Galia, Philip Robinson, Arnaud Fernandez, Florence Askenazy, Nicolas Chauliac, Philippe Vignaud

**Affiliations:** ^1^Centre Régional du Psychotraumatisme, Hôpital Edouard Herriot, Hospices Civils de Lyon, Lyon, France; ^2^Cellule d’Urgence Médico-Psychologique, Hôpital Edouard Herriot, Hospices Civils de Lyon, Lyon, France; ^3^Direction de la Recherche en Santé, Hospices Civils de Lyon, Lyon, France; ^4^Service Universitaire de Psychiatrie de l’Enfant et l’Adolescent, Hôpital pédiatrique Lenval de Nice, Nice, France

**Keywords:** intimate partner homicide, femicide, guidelines, protocol, child

## Abstract

In intimate partner homicides, children are confronted with multiple losses and become simultaneously a victim and the child of a murderer. These homicides have a very negative effects of these tragedies on these children There is a need to provide them early care, and this requires straightforward guidelines. The objective is to assess the feasibility of implementing such a feminicide protocol and to discuss, point-by-point, the difficulties of their application. It includes a series of 17 steps, from the commission of the offense to the end of 72 h hospitalization. Data regarding the completion of steps was to be collected for each of these situations. During the study period there were 4 intimate partner homicides; these involved 14 children. Overall, the protocol criteria were applied at a rate of 88.6%, 9/17 criteria where applied for each child. However, certain provisions, including a shorter duration of hospitalization, the absence of personal belongings, the lack of hearing the child witnesses and, above all, the restriction of visiting rights during hospitalization, are worth noting. Operational deviations of the protocol from the theoretical version are discussed. The present study reported encouraging results concerning the feasibility of the “femicide protocol” with the co-victim children, but the discrepancies between the protocol and the implementations reported in that study require to reflect about optimizations of the protocol and their potential influence on children’s wellbeing. The recent extension of the protocol in the French national territory will provide the professionals concerned with opportunities to solve the remaining challenges.

## Highlights

- Femicide is a tragedy with great traumatic potential for the children who are its co-victims.
- The care of these children requires a structured multidisciplinary protocol with a 72-h hospital stay.
- Femicide protocol can be applied, with the adjustment of certain criteria, to children co-victims of intimate partner homicide.

## 1 Introduction

Around 1 million homicides are committed each year across the world. Among these, 13.5% are thought to be intimate partner homicides and most victims are women; furthermore, more than a third (39%) of homicides of women are due to intimate partner violence while this represents only 6% of homicides of men (1). In these situations, children are confronted with multiple losses and become simultaneously a victim and the child of a murderer. Not only are there two parental attachment figures brutally taken from them, but they are also removed from their familiar environment. They cope with extreme violence, and face grief, stigma, and loyalty conflicts (2). The psychological trauma can lead to the occurrence of developmental and psychological disorders and may result in a psychological trauma that can lead to helplessness in the face of extreme danger, violence, and anxiety. Although not all DSM-5 criteria are always met, psychotraumatic symptoms are the most frequently described (3–6). In addition, other psychiatric complications are also reported, such as mood disorders and depression that can lead to suicidal thoughts and behavioral problems (2, 7–9). Moreover the children exposed to such violences have a higher risk to suffer from an unsecure attachment (10), or from problems with perceptual and cognitive functioning (11), to face academic difficulties, and interpersonal difficulties (12).

Recently studies conducted in many countries clearly show not only the very negative effects in terms of psychiatric disorders of these tragedies on the children who are co-victims, but also the need to provide them early care (2, 13, 14). However, up to now, no specific procedure providing these children with adequate care and an early support to prepare the best form of custody has been reported in the current literature. This is of importance as children are often immediately entrusted to the maternal grandmother, who is in mourning for her daughter, or to the paternal grandmother, the murderer’s mother or even an aunt (the mother’s or father’s sister), who may herself be a victim of intimate partner violence. In other words, when a tragedy occurs to a child, the family usually represents a protective environment, but in this type of event the family is dysfunctional. The question of a psychological evaluation of the child as well as the environment in which the child will be placed is therefore essential in these situations. As some authors recommend, this requires straightforward guidelines which means a commitment from all professional stakeholders, within a framework of precise rules and anticipated in the form of a protocol (15). This was implemented in a metropolitan area (located in southeastern of France, a conurbation of about 1.3 million inhabitants) through a “femicide protocol” signed on April 27, 2021 by all the partners involved. This protocol comprises various steps to ensure immediate hospitalization of children for 72 h. These steps have been declined into 17 criteria to assess the feasibility of such a protocol and to discuss, point by point, the difficulties of their application herein.

## 2 Methods

### 2.1 Procedure

Numerous meetings were organized between all the stakeholders concerned by intimate partner homicide (judicial, emergency services, health and social services) in order to develop the “femicide protocol” for the care of children following this event. The protocol was designed in order to reach the following goals: (i) providing the involved children with a safe place in this emergency context; (ii) providing the involved children with a reassuring and quite neutral emotional atmosphere. Indeed both parts of the family may get very affected by the femicide: the mother’s family is bereaved; the father’s family is facing a very challenging loyalty conflict; (iii) providing the professionals involved (judicial, emergency services, health and social services) with neutral and adequate conditions to complete their investigations; (iv) avoiding the interview with the police investigator to get impaired by the environment; (v) assessing very early the psychological state of the involved children, especially whether they suffer from an acute stress disorder. Thus, the protocol included 17 steps targeting these goals (they are listed in the Results section). The key element of this protocol is the immediate protection of the minors concerned through emergency hospitalization for 72 h. It was made possible by a judicial authority who drove the process and attempted to find a solution to the various problems encountered in these situations. The professionals and institutions involved in the protocol implementation are the following ones: medical emergency unit, judicial authority, emergency department, pediatric hospital (or at least pediatric department), liaison psychiatry, medico psychological emergency unit, social service, police investigation department, social services. This “femicide protocol” was signed on April 27, 2021.

### 2.2 Study design and participants

For the present retrospective study using data collected prospectively, all children concerned by intimate partner homicide occurring from the signing of the agreement (April 27, 2021) to the end of 2023 (December 31, 2023) in the concerned area were included. Data regarding the completion of 17 steps was to be collected within 8 days following the event using a paper-based form (yes, no, not applicable) and from review of the legal and clinical files for each of these situations. Non-applicable means that a criterion cannot be applied in this situation. In case of missing data, the professionals concerned by the case could be contacted for further information, and when a criterion was not applied the reasons for this were sought from the same sources. This study was approved by the local hospital’s ethics committee.

### 2.3 Analysis

Descriptive statistics were used to analyze the data. The analysis included presenting the values (n) when the “femicide protocol” was applied, the total number of children (N), as well as the corresponding percentages (%).

## 3 Results

During the study period there were 4 intimate partner homicides; these involved 14 children who were all included in the present study and all study data was collected from legal and clinical files within 8 days following the event for all children. These were mostly minors (13/14) who were mostly present at the time of the homicide (11/14) (Table 1).

**Table 1:** Presentation of the four situations.

Situation	Number of siblings	Children (<18 years old)			Adults (≥18 years old)	
		Number (age in years)	Present in the home at the time of the homicide (age in years)	Not present at the time of the homicide	Number	Present in the home at the time of the homicide (age in years)
1	5	3 (4, 9, 12)	3	0	2	0
2	4	4 (4, 6, 10, 13)	3	1 (13)	0	0
3	3	2 (9, 17)	1	1 (17)	1	1 (19)
4	4	4 (5, 8, 11, 13)	4	0	0	0

Overall, the protocol criteria were applied at a rate of 88.6%, 9/17 criteria where applied for each child (100%; criteria 1, 2, 3, 4, 13, 14, 15, and 16). Three criteria where applied at a minimum of 90% and below 100% (criteria 5, 8, and 9). In situation 1, the youngest child, who was present at the scene, was not hospitalized with the rest of the siblings (criterion 5). In the clinical file it was indicated that this was because the judicial authority present at the scene did not have the courage to remove the child from the maternal aunt’s arms therefore could not have somatic and psychiatric evaluation within 72 h (criteria 8, 9). In situation 1, this is also the reason why the youngest child was not hospitalized for 72 h (criterion 11) and separated from the siblings (criterion 7). Four criteria where applied between 69.2 and 78.6% (criteria 6, 7, 11, and 17). In situation 3, both children were hospitalized only 48 h (criterion 11). It was indicated in the clinical file that the care team agreed that the situation was managed and resolved for the children after 2 days in hospital. In situation 2, the children could not be interviewed by the investigators during their hospital stay (criteria 17). The reasons given in the file were organizational problems on the part of the police personnel who were obliged to postpone the interview. In situation 4, none of the children had access to their personal belongings (clothes, comforter, usual games, etc.; criterion 6). It was indicated in the clinical file that a young child psychiatrist blocked the transfer of the children’s belongings at the hospital, fearing that they would be stained with blood of the homicide and could reactivate the traumatic scene. The least frequently fulfilled criterion was criterion 12, i.e., the suspension of visits to children during hospitalization (33.3%). With the exception of the 4 children in situation 4, in which the medical team considered that contact with family members was not appropriate, visiting rights were not suspended during the 72 h of hospitalization. It was indicated in the clinical files that there were few visits to the children and were the subject of concerted discussion within the care team. The results are summarized in Table 2.

**Table 2:** Evaluation of the protocol application.

Criterion		Situation 1					Situation 2						Situation 3					Situation 4						n/N	%
	Years old	4	9	12	n/N	%	4	6	10	13	n/N	%	9	17	19	n/N	%	5	8	11	13	n/N	%		
	Present in the home at the time of the homicide	Y	Y	Y			Y	Y	Y	N			Y	N	Y			Y	Y	Y	Y				
1	Protocol triggered by the on-duty magistrate	Y	Y	Y	3/3	100	Y	Y	Y	Y	4/4	100	Y	Y	Y	3/3	100	Y	Y	Y	Y	4/4	100	14/14	100
2	The child was placed under the care of the ASE	Y	Y	Y	3/3	100	Y	Y	Y	Y	4/4	100	Y	Y	n.a.	2/2	100	Y	Y	Y	Y	4/4	100	13/13	100
3	The EMS notified the emergency pediatrician and child psychiatrist on duty at the corresponding hospital	Y	Y	Y	3/3	100	Y	Y	Y	Y	4/4	100	Y	n.a.	Y	2/2	100	Y	Y	Y	Y	4/4	100	13/13	100
4	The EMS contacted the CUMP on-call personnel	Y	Y	Y	3/3	100	Y	Y	Y	Y	4/4	100	Y	Y	Y	3/3	100	Y	Y	Y	Y	4/4	100	14/14	100
5	The child was transported to hospital ER	N	Y	Y	2/3	66.7	Y	Y	Y	n.a.	3/3	100	Y	n.a.	Y	2/2	100	Y	Y	Y	Y	4/4	100	11/12	91.7
6	The child had his personal belongings with him/her	Y	Y	Y	3/3	100	Y	Y	Y	Y	4/4	100	Y	n.a.	Y	2/2	100	N	N	N	N	0/4	0	9/13	69.2
7	Siblings were not separated	N	N	N	0/3	0	Y	Y	Y	Y	4/4	100	Y	Y	Y	3/3	100	Y	Y	Y	Y	4/4	100	11/14	78.6
8	The child underwent a somatic evaluation in the ER within 72 hours	N	Y	Y	2/3	66.7	Y	Y	Y	Y	4/4	100	Y	n.a.	Y	2/2	100	Y	Y	Y	Y	4/4	100	12/13	92.3
9	The child underwent a psychiatric evaluation in the ER within 72 hours	N	Y	Y	2/3	66.7	Y	Y	Y	Y	4/4	100	Y	n.a.	Y	2/2	100	Y	Y	Y	Y	4/4	100	12/13	92.3
10	The child underwent a psychotraumatic evaluation by the CUMP within 72 hours	Y	Y	Y	3/3	100	Y	Y	Y	Y	4/4	100	Y	Y	Y	3/3	100	Y	Y	Y	Y	4/4	100	14/14	100
11	The child was hospitalized at least 72 hours	N	Y	Y	2/3	66.7	Y	Y	Y	Y	4/4	100	N	n.a.	N	0/2	0	Y	Y	Y	Y	4/4	100	10/13	76.9
12	All visiting rights were suspended for 72 hours	n.a.	N	N	0/2	0	N	N	N	N	0/4	0	N	n.a.	N	0/2	0	Y	Y	Y	Y	4/4	100	4/12	33.3
13	The medical assessment was transmitted to the ASE	n.a.	Y	Y	2/2	100	Y	Y	Y	Y	4/4	100	Y	n.a.	Y	2/2	100	Y	Y	Y	Y	4/4	100	12/12	100
14	The hospital physician informed the ASE of the expected date of hospital discharge	n.a.	Y	Y	2/2	100	Y	Y	Y	Y	4/4	100	Y	n.a.	Y	2/2	100	Y	Y	Y	Y	4/4	100	12/12	100
15	The ASE assessed the child within 72 hours	Y	Y	Y	3/3	100	Y	Y	Y	Y	4/4	100	Y	Y	Y	3/3	100	Y	Y	Y	Y	4/4	100	14/14	100
16	The ASE sent its assessment report to the public prosecutor and made a proposal for a suitable place to accommodate the child when he or she left hospital	Y	Y	Y	3/3	100	Y	Y	Y	Y	4/4	100	Y	Y	Y	3/3	100	Y	Y	Y	Y	4/4	100	14/14	100
17	Investigators interviewed the child during hospital stay	Y	Y	Y	3/3	100	N	N	N	N	0/4	0	Y	Y	Y	3/3	100	Y	Y	Y	Y	4/4	100	10/14	71.4
																								Mean	88.6

Y, Yes; N, No; n.a., not applicable (cannot be applied in this situation); EMS, Emergency Medical Service; CUMP, *Cellule d’Urgence Médico-Psychologique* (Medical and Psychological Emergency Unit); ASE, *Aide Social à l’Enfance* (Child Welfare Services); ER, Emergency Room.

## 4 Discussion

The “femicide protocol” was mostly applied to the 14 children in the 4 intimate partner homicides that occurred during the study period. Taking these results together, we can therefore consider this protocol applicable which justifies its continuation in order to provide better care for children who are co-victims of these tragedies. This is in line with the scientific literature, which stresses the short, medium and long-term psychological consequences of these events for children (2, 7–9) and recommends codified procedures to be followed after such event (15). The present study is also of particular interest as the scope of the “femicide protocol” was expanded, without prior evaluation, nationally through a circular on April 12, 2022 (Instruction N° DGOS/R4/DGCS/PEA/2022/103). However, certain provisions, including that a very young child was not hospitalized, a shorter duration of hospitalization for certain children, the absence of personal belongings for the children, the lack of hearing the child witnesses during the hospitalization, and, above all, the restriction of visiting rights during hospitalization, are worth noting.

### 4.1 Reasons why criteria were not applied and proposals for improvement

#### 4.1.1 Concerted motives

Based on the clinical records, some of the criteria not applied appeared to be the result of a concerted decision by the care team. The 48-h hospital stay for one sibling was an exception, justified by the fact that the children’s care was complete at the end of this period. Considering all the steps included in the protocol, one may get surprised that there were completed so quickly; the anticipated end of the hospitalization may also have relied on an hospital overcrowding, a recurrent challenge in the public health system. Theoretically, the femicide protocol was designed with an at least 72-h hospital stay, in order to complete every steps. The psychological burden caused by an hospital stay was investigated and should be taken in consideration; nevertheless this risk seems to target clearly more prolonged and/or recurrent hospitalizations than acute and short ones (16–18).

The criterion 12 (suspension of visiting rights) was a recurrent failure in the implementation of the protocol. From the protocol’s perspective, the measure aims at protecting the involved children by preventing interactions with relatives who are also probably directly or indirectly affected by the homicid and may not be able to provide these children with an adequate emotional support. Moreover this measure also involves judiciary and social issues; indeed restricting visiting rights notably decreases the risk that the investigator interview gets influenced by external factors. However, despite this rationale, the criterion 12 should be discussed. Indeed, not only some authors reported that an hospital stay could induce a negative psychological impact (16, 18) but several sources of comfort were described in previous studies in order to prevent it such as visits by parents, visitors and friends (19). Although a full suspension of visits is feasible (as reported in the situation 4), the failure to comply with this provision, which was mostly supported by a collaborative decision of the care team and concerned the majority of children, highlights the difficulty of completely isolating children from their relatives. Future studies investigating the protocol’s impact will have to deal with this specific criterion and its possible implementations in routine activity: no visit, no restriction to visits, partial restriction. The criterion 6 (the child had his/her personal belongings with him/her) was another recurrent failure. Not only it is a part of the protocol’s design but it is also supported by the literature as belongings such as the stuffed toy were reported to be sources of comfort during an hospital stay (19). So it appears as an important criterion that should not be ignored. Yet, in some cases, the personal belongings might reactivate the traumatic scene (as an example a stuffed toy stained with blood). In order to improve its implementation, a possible adaptation of the criterion 6 would consist in selecting on a subtler way the personal belongings the children are transported to hospital with, implying to standardize the choice and the factors allowing some exceptions.

#### 4.1.2 Personal or organizational factors

Other criteria of the protocol that were not implemented were clearly due to personnel and/or organizational factors. The 4-year old child, immediately taken away by her aunt without the judicial authorities present at the scene preventing it, separated from the sibling and therefore excluded from important initial provisions, highlights both the lack of, and the need for, training of the professionals involved who may themselves be deeply affected in these. Another example of a failure to comply with the protocol was the refusal to bring to the hospital the personal belongings for the children, on the grounds that they might reactivate the traumatic scene, and despite the fact that these criteria were widely recommended in the protocol. This reflects the need to train young child psychiatrists both in the value of this protocol and in the clinical aspects of psychotrauma. Another failure to comply with the protocol, due to a lack of organization on the part of the investigators, meant that the children’s interviews had to be postponed until after the hospital discharge, which was harmful for them as they were no longer in a care environment. This also indicates that investigators need to be made aware of the importance of conducting the interviews in a protected environment.

### 4.2 Further considerations

It is of note, that in one situation reported herein, a 19-year-old was involved in the protocol (situation 3), as did the siblings, which, in hindsight, seemed particularly important. This suggests that the protocol should be extended to include young adults. Moreover, it may also be interesting to further extend the protocol to minors present at the scene but not related to the couple. This has yet to arise, but could be beneficial for the children involved, but would require adaptation of the protocol, in particular visits and evaluation by child welfare services. In addition, it is important to ensure that this protocol is also applied in exceptional cases where the victim is a man killed by his spouse.

### 4.3 Strengths and limitations

To the best of our knowledge, our article is the first report dealing with a standardized protocol targeting the children involved in a femicide. Although this is an uncommon event, its highly negative psychological and psychiatric impact on the children co-victims justify to design specific tools to face this challenge.

Nevertheless, several limitations have to be highlighted. First, the main limitation of the present study is that it included only a small number of children, despite the exhaustiveness of the inclusion period, but which is due to these events being, fortunately, infrequent. Further multicenter studies involving a larger population would enhance the strength of conclusions. The recent extension of the protocol on the whole French national territory will support the implementation of such studies.

A second significant limitation of the study is a lack of standardized psychometric scales and qualitative psychologic assessments of the involved children. This was not any goal of our study, which rather targeted a feasibility evaluation. Future studies about this feminicid protocol will have to incorporate validated psychometric assessments, yielding a more comprehensive understanding of its impact.

Last, as reported above, the failure to apply some criteria was repeatedly related to a “concerted decision” of the care team. This decision process influenced by the clinician experience should be considered as strength and weakness as well. On the one hand, one may consider that the protocol allows a notable degree of freedom. This is a precious feature since some flexibility seems required for this protocol as its trigger (namely the femicide) is both rare and emotionally challenging for the professionals involved. On the other hand, these *ad hoc* decisions weaken the standardization process promoted by the protocol, leading to supplementary barriers in its generalization. In order to improve its standardized implementation and/or its ergonomics, further studies on the femicide protocol should investigate the following concerns: (i) the priority level of the different criteria; (ii) the permissible deviations for the criteria that cannot be applied and/or that do not seem mandatory.

It is therefore necessary to continue the evaluation of the protocol, and as this aims, above all, to treat the child co-victims further studies are also needed on the long-term assessment of the children concerned.

## 5 Conclusion

The present study reported encouraging results concerning the feasibility of the “femicide protocol” with the co-victim children. Yet, some failures in the implementation of specific criteria should be noted. Further studies about this protocol are required and they will have to investigate several concerns: (i) the priority level of the different criteria; (ii) the implications when a specific criterion is not implemented; (iii) the permissible deviations to the theoretical protocol; (iv) including a validated psychological assessment in the children included in the protocol, and (v) the support which should be provided to the involved professionals in order to help them to implement the protocol as well as possible. Such investigations will be facilitated by the extension of the protocol to the whole French national territory 2022.

## Data Availability

The original contributions presented in the study are included in the article/supplementary material, further inquiries can be directed to the corresponding authors.
